# Supplementary material for: Accuracy of Intra-Axial Brain Tumor Characterization in the Emergency MRI Reports: A Retrospective Human Performance Benchmarking Pilot Study
Source: Diagnostics (Basel). 2024 Aug 16;14(16):1791. doi: 10.3390/diagnostics14161791 (PMC11353410; doi:10.3390/diagnostics14161791)
Supplement: Supplementary file 1 [file diagnostics-14-01791-s001.zip › diagnostics-3108430-supplementary.pdf]

Table S1. Acquisition parameters for MRI sequences.

	**Axial T2-weighted TSE**	**Axial T2 FLAIR**	**Isotropic 3D FLAIR**	**Axial DWI**	**Axial SWI**	**Isotropic 3D TSE T1**	**Isotropic 3D TSE T1 with gadolinium***
TR (ms)	4251.5	11000	4800	4625	18	600	600
TE (ms)	80	120	310	87	25	28	28
TI (ms)	–	2800	1650	–	–	–	–
Flip angle	90	90	90	90	15	90	90
Slice thickness (mm)	3	5	1.12	4	2	1	1
Slice spacing	4	6	0.56	4	1	0.5	0.5
Matrix size	512 x 418	240 x 179	224 x 224	152 x 432	256 x 256	252 x 249	252 x 249

TR Repetition time, *TE* Echo time, *TI* Inversion time.
*Dotarem (Guerbet, France) 279.3 mg/ml, 20 ml intravenous injection.
